# The Effect of interventions based on behavioral change modeling in traffic injuries ‎prevention: a review Study 

**Published:** 2019-08

**Authors:** Morteza Haghighi, Fatemeh Bakhtari Aghdam, Mohammad Saadati

**Affiliations:** ^*a*^Road Traffic Injury Research Center, Health Management and Safety Promotion Research Institute, Tabriz University of Medical Sciences, Tabriz, Iran.

## Abstract

**Background:**

Behavioral change models in health education and health promotion are used to create and ‎modify various behaviors. The purpose of this study is to examine patterns used in traffic and ‎promote safe behaviors.‎

**Methods:**

In this overview, databases including PubMed, Medline, Since Direct, SID and Google scholar ‎were searched using the keywords pedestrians, drivers, riders, traffic, safe behaviors, models ‎Health promotion, health education, intervention and behavior change among the articles ‎published in last 10 years. ‎

**Results:**

After the final review of the retrieved articles, 36 studies based on health promotion models ‎‎(Both interventional and non-interventional) were included which was 18 for Iran. The most ‎used model was the theory of planned behavior (25 cases) and then the health belief model (5 ‎cases). In this study, the target group was 19 drivers, 3 were motorcyclists and 14 were ‎pedestrians. In driving studies, more health education models have been used to utilize seat ‎belts and distraction factors and health education models were used more often in motorcyclists ‎to use helmets and pedestrians in relation to safe behaviors. ‎

**Conclusions:**

If health education and health promotion models are used effectively to change behavior, it is ‎possible to reduce crashes and create safe behaviors for all road users.‎

**Keywords:**

Model-Based interventions, Traffic, Review study

